# Non-puerperal Breast Actinomycosis: A Case Report

**DOI:** 10.7759/cureus.42092

**Published:** 2023-07-18

**Authors:** Miriam Sousa, Ana Pereira, José Alves, Marília Gonçalves, Ana Vicente

**Affiliations:** 1 Pathology, Hospital Central do Funchal, Funchal, PRT; 2 Radiology, Hospital Central do Funchal, Funchal, PRT

**Keywords:** maldi-tof ms, actinomyces neuii, actinomyces spp, actinomycosis, breast abscess, mastitis

## Abstract

Breast abscesses can be a complication of infectious mastitis or the first presentation of a breast infection, presenting as pain, erythema, and a lump. Actinomycosis is a rare chronic disease caused by anaerobic branched filamentous gram-positive bacteria belonging to the *Actinomyces* genus. It is usually found in the human mouth, digestive tract, and genital tract but can also cause breast abscesses. Actinomycosis affecting the breast is a rare condition that typically occurs as a secondary infection resulting from a pulmonary infection. It is primary when inoculation occurs through the nipple. This report describes the case of a 48-year-old institutionalized female with periareolar swelling in the right breast that had been evolving for approximately two months. The abscess was drained, and the aspirated material contained *Actinomyces neuii*, a gram-positive bacterium. Actinomycosis of the breast can manifest as either a sinus tract or mass-like features that closely resemble malignancy. The clinical presentation can pose challenges in distinguishing between primary actinomycosis, mastitis, and inflammatory carcinoma. Treatment consists of drainage with needle aspiration or surgical drainage and antibiotic therapy.

## Introduction

Mastitis is a relatively common breast condition that can affect patients at any time; however, it is predominant in women during the breastfeeding period [1]. In general, mastitis is an inflammation of the breast that may or may not be accompanied by infection [2]. Mastitis can be classified into three main groups: infectious, noninfectious, and malignant [2].

A breast abscess is a localized collection of purulent material in the breast tissue, which can be a complication of infectious mastitis (developing in 3-11% of women with mastitis) or can also be the first presentation of breast infection [1-3]. It is important to acknowledge that several other pathologies can exhibit similar symptoms to a breast abscess. The differential diagnosis for a breast abscess encompasses various infectious conditions, malignancies, traumas, dermatologic conditions, venous and lymphatic abnormalities, as well as idiopathic causes [4].

Breast abscesses primarily occur in women aged between 18 and 50 years. Among women of reproductive age, lactational abscesses are more prevalent, but non-lactational abscesses can also occur in premenopausal older women [1]. Non-lactational abscesses are more common in obese patients and smokers than in the general population [1]. Non-lactational breast abscesses are relatively uncommon compared with lactational abscesses. Approximately 90% of non-puerperal breast abscesses are subareolar. The remaining non-lactational breast abscesses are caused by rare granulomatous, bacterial, or fungal etiologies [4].

Bacterial colonization of the skin is the primary cause of infectious mastitis and breast abscesses. The most prevalent causative agent is *Staphylococcus aureus*, with coagulase-negative Staphylococci being the second most common [1]. Some breast infections may be polymicrobial, and anaerobes are sometimes isolated from abscesses and chronic recurrent cases [1]. The most uncommon pathogens are *Bartonella henselae*, *Mycobacteria*, *Actinomyces*, *Brucella*, fungi, and parasites [3].

Breast actinomycosis is rare and usually occurs secondary to pulmonary infection [5]. Primary actinomycosis of the breast is extremely rare and occurs when the bacterium is inoculated through the nipple [6]. Possible causes of this condition include trauma, lactation, and kissing [7]. Most reported cases of actinomycosis of the breast involve pre-menopausal women [3].

Breast abscesses are typically diagnosed based on their clinical presentation and medical history, as they commonly manifest with symptoms such as pain and/or the presence of a palpable lump in the breast. Physical examination usually identifies pain, erythema, and firmness over an area of the breast at the abscess location. Imaging studies may assist in the diagnosis of breast abscesses [4]. Ultrasound is the preferred imaging modality, and fine-needle aspiration can be used to drain a breast abscess for diagnostic and therapeutic purposes [1].

Primary breast abscess management involves drainage using needle aspiration or surgical drainage and antibiotic therapy [3].

## Case presentation

A 48-year-old institutionalized female with a history of psychiatric disorders, hypertension, and smoking habits presented to the emergency department with periareolar swelling in the right breast that had been evolving for approximately two months. Physical examination revealed erythema and tenderness. Axillary lymphadenopathy was observed in the right arm of the patient. There was no history of fever, breast trauma, or signs or symptoms of any lung disease. The mass in the right breast was suggestive of a neoplasm; thus, mammography and ultrasonography were requested as a matter of urgency.

**Figure 1:**
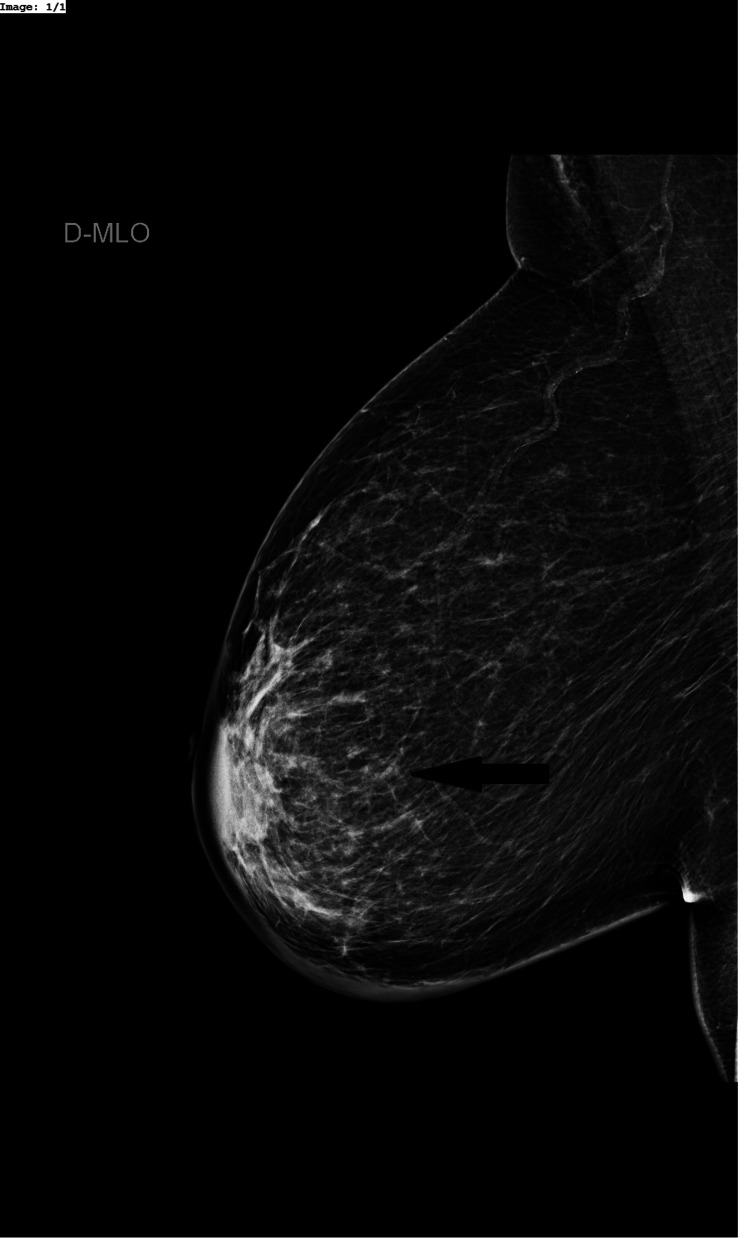
Right mammography

**Figure 2:**
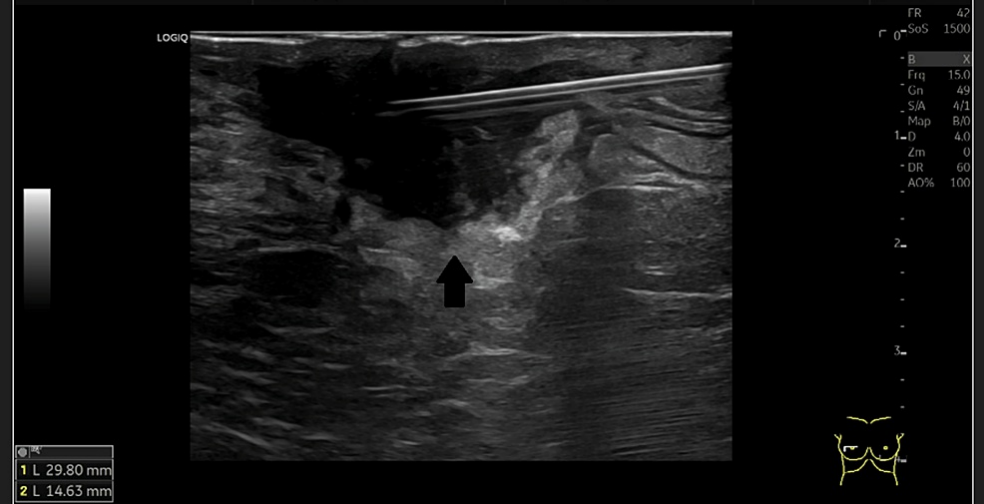
Irregular collection in the right breast

**Figure 3:**
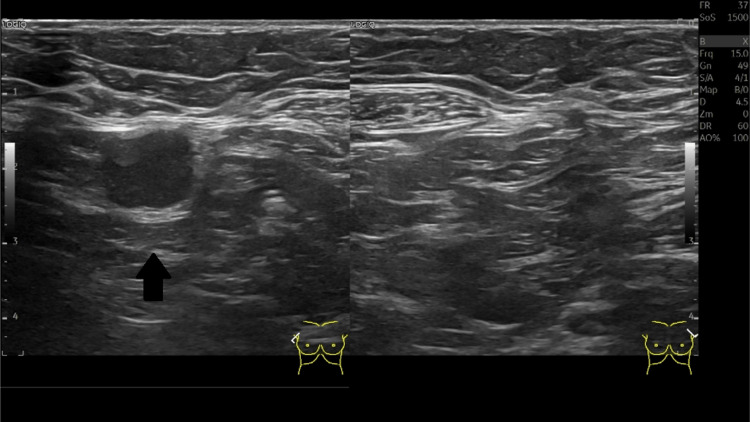
Ultrasonography of the right axillary lymphadenopathy

The mammography revealed an increased density of the right perimammary cutaneous tissue, corresponding to a palpable lump (Figure 1). The ultrasonography of the right breast showed an irregular collection with thickened walls. It measured approximately 30 mm of the greatest axis (Figure 2). A 14 mm adenopathy was observed in the right axilla (Figure 3). The abscess was drained using a 19G needle, and the aspirated material was sent to the laboratory for microbiological studies, which showed evidence of pus (Figure 3). An axillary biopsy was not performed because it was considered reactive to the abscess.

Gram stain showed many leukocytes and many Gram-positive rods. After two days, colonies were visible in an anaerobic atmosphere on a blood agar plate. Identification using matrix-assisted laser desorption ionization-time-of-flight mass spectrometry (MALDI-TOF MS; Bruker) revealed *Actinomyces neuii*, a gram-positive bacterium. Susceptibility testing for *Actinomyces neuii* was performed using disk diffusion according to the European Committee on Antimicrobial Susceptibility Testing recommendations, and *Actinomyces neuii* was found to be susceptible to penicillin, amoxicillin, clindamycin, and cephalosporin. Subsequently, the patient was treated with prolonged antibiotic therapy with amoxicillin and clavulanic acid and was reevaluated for three months.

## Discussion

Actinomycosis is a rare chronic disease caused by *Actinomyces* spp., an anaerobic gram-positive bacterium that typically colonizes the mouth, digestive tract, and genital tract of humans [8]. The disease usually presents as cervicofacial, thoracic, abdominal, or pelvic infection [5] and commonly exhibits as a chronic, slowly progressing infection characterized by the development of abscesses [9]. It frequently mimics malignancy, tuberculosis, or nocardiosis [8].

*Actinomyces* is a diverse group of gram-positive bacteria, primarily composed of facultatively anaerobic and microaerophilic rods, exhibiting varying degrees of branching [3]. Over 30 *Actinomyces* spp. have been documented in scientific literature. *Actinomyces* infections can be polymicrobial and often involve other bacteria that play a role in initiating and progressing the infection by hindering host defenses or reducing oxygen levels. Most *Actinomyces* spp. are facultative anaerobes, necessitating anaerobic incubation conditions for culture. Chocolate blood agar media at 37 °C is commonly used for *Actinomyces* cultivation, along with other enriched media, such as brain heart infusion broth and *Brucella* blood agar supplemented with hemin and vitamin k1. The initial suspicion of *Actinomyces* was based on colony morphology and biochemical profiling. Traditional identification methods rely on phenotypic tests including urease, catalase, and fermentation of sugars. However, MALDI-TOF MS is a faster and more accurate tool for *Actinomyces* identification. This technique employs mass spectrometry, which involves the ionization, separation, and detection of ionized bacterial proteins to generate a species-specific mass spectrum [8].

All rare cases of primary actinomycosis were caused by *Actinomyces israelii*, the most common *Actinomyces* associated with human infections (approximately 80 %) [6-9]. The species *Actinomyces neuii* is less well known to cause classical actinomycosis and may not cause classical actinomycosis [5-9]. Among all clinical *Actinomyces* isolates, *Actinomyces neuii* was detected in 17% of cases. This particular species distinguishes itself from other *Actinomyces* species due to its ability to grow aerobically and its distinctive microscopic morphology lacking branching structures [9]. *Actinomyces neuii* exhibits a preference for specific regions of the human body as an infectious agent. Among the infections from which *Actinomyces neuii* has been isolated, abscesses and infected atheromas are the most commonly observed. The majority of abscesses were localized in the mammary, axillary, and inguinal areas, mirroring the pattern seen in other *Actinomyces* spp. In a minority of abscesses and atheromas, *Actinomyces neuii* was found in pure culture, with the majority yielding mixed anaerobic conditions [5].

Bacterial cultures and histopathological examination are the cornerstones of diagnosis [8]. *Actinomyces* growth is slow and incubation for at least 10 days is required before concluding a negative culture [8]. Culture is positive only in a minority of cases and remains sterile in more than 50% of cases [8]. Gram stain is usually more sensitive than culture in establishing a diagnosis. In direct Gram staining, *Actinomyces neuii* appears to be a diphtheroid and even a coccoid organism without branching [5]. The culture failure rate is elevated due to various factors, including prior antibiotic treatment, the suppression of *Actinomyces* growth by concurrent or contaminating microorganisms, inadequate culture conditions, and insufficient short-term incubation periods [8]. In our case, histopathological examination was not performed.

Actinomycosis is generally not associated with drug resistance. *Actinomyces* spp. are typically highly susceptible to beta-lactam antibiotics, particularly penicillin G or amoxicillin [8]. The antimicrobial susceptibility of *Actinomyces neuii* corresponded to that of other *Actinomyces* spp. [5]. Prolonged antibiotic therapy with penicillin is the preferred treatment [7]. Patients diagnosed with actinomycosis typically require high doses of penicillin G or amoxicillin for a prolonged period, ranging from six to 12 months. This extended treatment duration is necessary to ensure effective drug penetration into the abscesses and infected tissues. However, if the infected tissues are optimally removed through surgical resection, the duration of antimicrobial therapy may be reduced to three months [8]. Implementing specific preventive measures such as reducing alcohol abuse and tobacco use and practicing good dental hygiene can help minimize the incidence of actinomycosis [8].

## Conclusions

Breast abscesses can be caused by *Actinomyces *spp., a rare chronic disease that usually colonizes the human mouth, digestive tract, and genital tract and can present as a sinus tract or as mass-like features that mimic malignancy, making it difficult to distinguish between primary actinomycosis, mastitis, and inflammatory carcinoma. To accurately diagnose actinomycosis, clinicians should communicate their suspicion to the microbiologist to ensure that the culture is performed under the necessary conditions. If actinomycosis presents as a well-defined abscess, percutaneous drainage combined with prolonged antibiotic therapy is a reasonable approach.

We can conclude that only a limited number of such cases have been documented in scientific literature. Therefore, this particular case is significant, as we now have access to new identification methodologies, such as MALDI-TOF MS, which enables the identification of uncommon microorganisms.

## References

[1] Boakes E, Woods A, Johnson N, Kadoglou N (2018). Breast infection: a review of diagnosis and management practices. Eur J Breast Health.

[2] Kamal RM, Hamed ST, Salem DS (2009). Classification of inflammatory breast disorders and step by step diagnosis. Breast J.

[3] (2022). Primary breast abscess. https://www.uptodate.com/contents/primary-breast-abscess.

[4] Lam E, Chan T, Wiseman SM (2014). Breast abscess: evidence based management recommendations. Expert Rev Anti Infect Ther.

[5] Gosavi AV, Anvikar AR, Sulhyan KR, Manek DD (2016). Primary actinomycosis of breast-A diagnosis on cytology. Diagn Cytopathol.

[6] Capobianco G, Dessole S, Becchere MP, Profili S, Cosmi E, Cherchi PL, Meloni GB (2005). A rare case of primary actinomycosis of the breast caused by Actinomyces viscosus: diagnosis by fine-needle aspiration cytology under ultrasound guidance. Breast J.

[7] Salmasi A, Asgari M, Khodadadi N, Rezaee A (2010). Primary actinomycosis of the breast presenting as a breast mass. Breast Care (Basel).

[8] Valour F, Sénéchal A, Dupieux C (2014). Actinomycosis: etiology, clinical features, diagnosis, treatment, and management. Infect Drug Resist.

[9] Leenstra BS, Schaap CC, Bessems M, Renders NH, Bosscha K (2017). Primary actinomycosis in the breast caused by Actinomyces neuii. A report of 2 cases. IDCases.

